# Development of a rabbit model for adrenoleukodystrophy: A pilot study on gene therapy using rAAV9

**DOI:** 10.1016/j.omtn.2025.102469

**Published:** 2025-02-03

**Authors:** Xiaoya Zhou, Chui-Yan Ma, Xiaoxian Zhang, Xianchuan Xu, Fuyu Duan, Meng Kou, Hongsheng Liu, Liang Zeng, Liyan Guo, Shaoxiang Chen, Li Chen, Ziyue Li, Jie Luo, Jieying Wu, Zhejin Li, Zhanjun Li, Tingting Sui, Ping Yuan, Zhijian Lin, Hao Chen, Liangxue Lai, Qizhou Lian

**Affiliations:** 1 Cord Blood Bank, Guangzhou Institute of Eugenics and Perinatology, Guangzhou Women and Children’s Medical Center, Guangzhou Medical University, Guangzhou 510623, China; 2 CAS Key Laboratory of Quantitative Synthetic Biology, Shenzhen Institutes of Advanced Technology, Chinese Academy of Sciences, Faculty of Synthetic Biology, Shenzhen University of Advanced Technology, Shenzhen 518055, China; 3 Center for Translational Stem Cell Biology, Hong Kong, China; HKUMed Laboratory of Cellular Therapeutics, University of Hong Kong, Hong Kong 999077, China; 4 Department of Surgery, The University of Hong Kong Shenzhen Hospital, Shenzhen 518053, China; 5 Department of Radiology, Guangzhou Women and Children’s Medical Center, Guangzhou Medical University, Guangzhou 510623, China; 6 Department of Pathology, Guangzhou Women and Children’s Medical Center, Guangzhou Medical University, Guangzhou 510623, China; 7 State Key Laboratory for Diagnosis and Treatment of Severe Zoonotic Infectious Diseases, Key Laboratory for Zoonosis Research of the Ministry of Education, Institute of Zoonosis, and College of Veterinary Medicine, Jilin University, Changchun 130062, China; 8 Guangdong Institute of Gastroenterology, Guangdong Provincial Key Laboratory of Colorectal and Pelvic Floor Disease, The Sixth Affiliated Hospital, Sun Yat-sen University, Guangzhou 510655, China; 9 Department of Neurology, Peking University Shenzhen Hospital, Shenzhen 518036, China; 10 Department of Gastroenterology, Guangdong Provincial People’s Hospital (Guangdong Academy of Medical Sciences), Southern Medical University, Guangzhou 510080, China; 11 CAS Key Laboratory of Regenerative Biology, Guangdong Provincial Key Laboratory of Stem Cell and Regenerative Medicine, Guangzhou Institutes of Biomedicine and Health, Chinese Academy of Sciences, Guangzhou 510530, China

**Keywords:** MT: RNA/DNA Editing, X-linked adrenoleukodystrophy, very long-chain fatty acids, ABCD1, rAAV9, gene therapy

## Abstract

X-linked adrenoleukodystrophy (X-ALD) is a common peroxisomal disorder caused by mutations in the *ABCD1* gene, leading to the accumulation of very long-chain fatty acids (VLCFAs). This progressive neurodegenerative disease manifests in three primary forms: childhood-acquired cerebral demyelination (CALD), adult myelopathy (AMN), and primary adrenal cortical insufficiency. Bone marrow transplantation effectively halts disease progression only in the early stages of CALD. A thorough investigation of the pathophysiology of X-ALD has been hampered by the lack of a reliable animal model. Valid animal models of X-ALD are urgently needed. To address this, we used CRISPR-Cas9 technology to knock out the *ABCD1* gene and established a novel rabbit model of X-ALD. The mutants exhibited elevated serum levels of hexacosanoic acid (C26:0), lignoceric acid (C24:0), and an increased C26:0/C22:0 ratio, as well as significant white matter demyelination in the brain and spinal cord. We also investigated rAAV9-based gene therapy in this model and found a significant reduction in VLCFAs. This study introduces CRISPR-Cas9-mediated *ABCD1* gene knockout rabbits as a novel animal model. It comprehensively evaluates the short-term outcomes and safety of rAAV-based gene therapy for X-ALD, providing a promising approach to explore the molecular and pharmacological mechanisms of the disease.

## Graphical abstract

**Figure:**
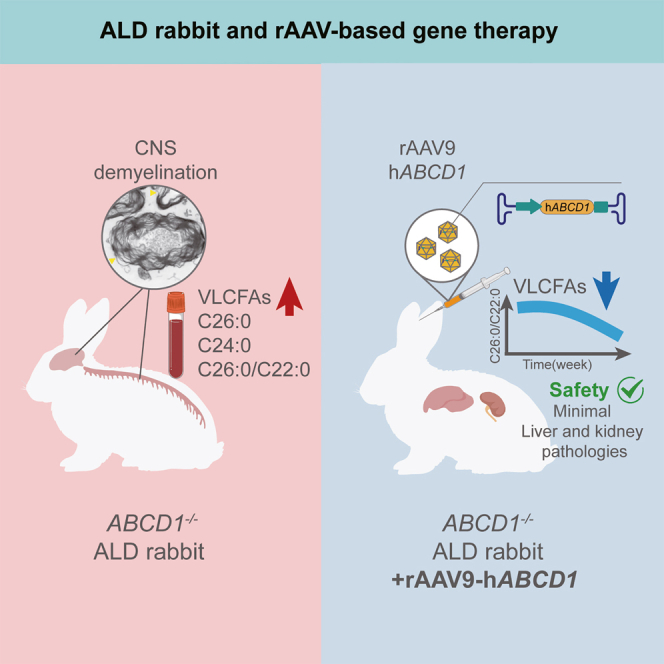

## Teaser

X-ALD is a globally prevalent hereditary metabolic disorder. CRISPR-Cas9-generated *ABCD1*^*−/−*^ rabbits exhibit elevated VLCFA levels and CNS demyelination, serving as a valuable model for X-ALD research. Lian and colleagues developed and preliminarily evaluated the efficacy and safety of rAAV9-based gene therapy.

## Introduction

X-linked adrenoleukodystrophy (X-ALD) is a recessive disorder linked to the X chromosome, caused by mutations in the ATP-binding cassette (ABC) subfamily D, member 1 (*ABCD1*) gene on Xq28.^1^^,^^2^ The adrenoleukodystrophy protein (ALDP or ABCD1) is critical for transporting very long-chain fatty acids (VLCFAs, ≥ C22:0) into peroxisomes for β-oxidation. Clinical studies show that ALDP preferentially transports saturated fatty acids to peroxisomes, such as lignoceric acid (C24:0) and hexacosanoic acid (C26:0).^3^ An elevated C26:0/C22:0 ratio in blood serves as a prominent diagnostic hallmark of X-ALD.^4^^,^^5^

X-ALD is categorized into three main types based on severity: rapidly progressive cerebral demyelination in childhood (CALD), progressive myelopathy in adulthood (adrenomyeloneuropathy, AMN), and primary adrenal cortical insufficiency.^2^^,^^6^ CALD is the most severe, affecting the central nervous system (CNS), with symptoms ranging from asymptomatic to severe disabilities or death.

Currently, there are no approved pharmacological treatments for AMN or CALD.^7^ While allogeneic hematopoietic stem cell transplantation (HSCT) is utilized for early-stage CALD, it does not prevent adrenal dysfunction and is limited by risks such as graft-versus-host disease and donor-matching challenges. Genetically modified autologous hematopoietic stem cell gene therapy (HSCGT) shows some efficacy in early-stage CALD and has slowed white matter degeneration in some cases.^8^^,^^9^ Nevertheless, 75% of patients still experience significant neurodegeneration.^10^ Lentivirus-based gene therapy, in which the lentivirus carrying the therapeutic gene is randomly inserted into the genome, provokes potential genomic toxicity. Modified elivaldogene tavalentivec (Lenti-D) has shown promise, but myeloablative chemotherapy side effects and long-term safety concerns need further study.^9^ Additionally, lentiviral vectors may take 6 months to cross the blood-brain barrier (BBB), during which irreversible deterioration can occur, indicating HSCGT is not yet a definitive cure for CALD, and its long-term clinical outcomes remain uncertain. Therefore, exploring alternative therapeutic strategies is necessary.

Recombinant adeno-associated virus serotype 9 (rAAV9) has demonstrated the ability to cross the BBB through intravenous injection and has shown partial symptom improvement in several CNS disease models, such as spinal muscular atrophy,^11^ Rett syndrome,^12^ and Alzheimer’s disease.^13^ Although adeno-associated virus (AAV)-based therapy has limitations, such as immunogenicity and the small size of the therapeutic gene, it offers several advantages. AAV-based therapy does not require integration into the genome, can cross the BBB, and provides lower costs and higher efficiency in mass manufacturing. These attributes make it advantageous in terms of safety and broad applicability. Food and Drug Administration (FDA)-approved AAV9-based gene therapies present a promising platform for treating various spinal cord and CNS neurodegenerative disorders.^14^

To better understand X-ALD pathophysiology, transgenic models, including *pmp-4*-deficient worms, *bgm dbb*-double knockout *Drosophila*, and *Abcd1*-deficient mice, have been developed.^15^^,^^16^^,^^17^^,^^18^^,^^19^^,^^20^ While the *pmp-4*-deficient worms and *bgm dbb* mutant *Drosophila* highlight axonal damage related to VLCFA and neural degeneration, they are not mammalian models, and the mutations are orthologues of *ABCD1*. *Abcd1*-deficient mice, which show elevated VLCFAs in tissue and blood along with myelopathy signs, are commonly used. Unfortunately, they do not exhibit spontaneous inflammatory demyelination.^21^^,^^22^ These models contribute to ALD research but fail to completely replicate the clinical symptoms of CALD, limiting accurate manifestations and predictions regarding drug reactions.^18^ The lack of a reliable X-ALD model hampers thorough investigations into the disease’s pathophysiology, indicating an urgent need for a preclinical X-ALD model. This study introduces *ABCD1* knockout (*ABCD1*^*−/−*^) rabbits (*Oryctolagus cuniculus*) as a novel X-ALD model, offering insights into the disease’s pathogenesis. Additionally, we developed rAAV9-based gene therapy to address *ABCD1* dysfunction in this new rabbit model and conducted a preliminary evaluation of treatment efficacy and safety.

## Results

### Generation of *ABCD1*-mutated rabbits via CRISPR-Cas9 gene editing

**Figure 1:**
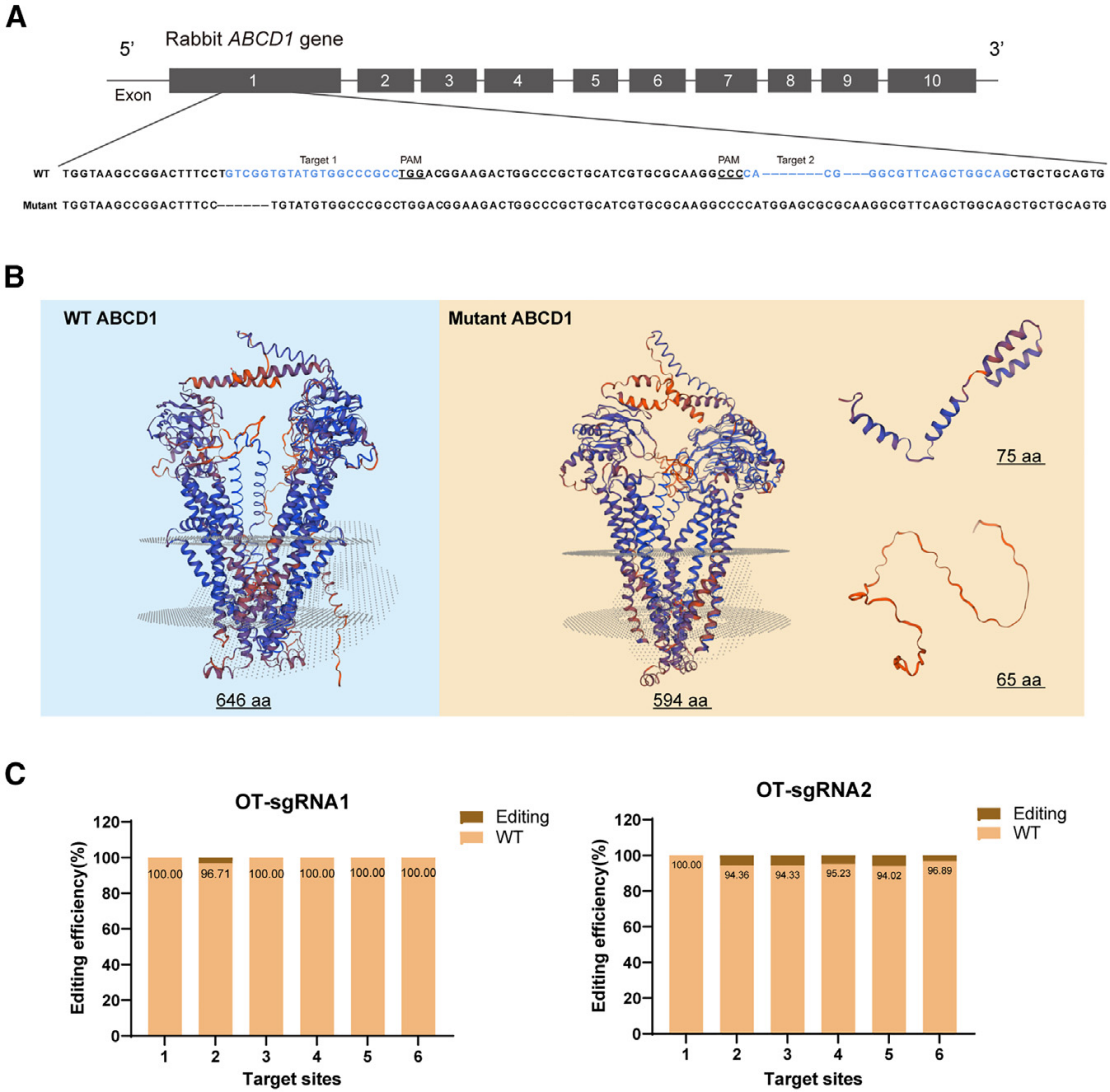
Mutation detection sequence and predicted protein structure of mutated ABCD1 (A) Sequencing information of *ABCD1*-mutated rabbits with two sgRNA targets highlighted in blue, and PAM sites underlined. (B) Predicted structure of WT ABCD1 and mutant truncated ABCD1. (C) Editing efficiency of sgRNA1 and sgRNA2.

Most pathogenic mutations occur in the transmembrane domain (exons 1 and 2), specifically within the first 300 amino acids (aa) of *ABCD1* exon1.^23^ Therefore, we designed two single guide RNAs (sgRNAs) targeting sequences within the first 300 aa of exon1. Cas9 mRNA and *ABCD1* exon1-specific sgRNAs were microinjected into rabbit zygotes, which were subsequently transferred into the oviduct of a female rabbit. This study encompassed four generations of *ABCD1*-mutated offspring, including heterozygotes (*ABCD1*^*−/+*^) and homozygotes (*ABCD1*^*−/−*^), which were bred from one editing zygote. Genotyping PCR and genomic DNA sequencing confirmed the mutations exclusively within exon1 of the *ABCD1* gene (Figure 1A).

To assess the impact of *ABCD1* knockout on protein expression, we used SWISS-MODEL to predict the structure of mutated ABCD1, yielding three truncated protein forms (594 aa, 75 aa, and 65 aa), while the predicted wild-type (WT) ABCD1 is 646 aa (Figure 1B), confirming the introduction of a premature stop codon.

Predicted off-targets were identified using a dedicated website (http://www.rgenome.net/cas-offinder/Sanger), selecting the top six potential sites to evaluate sgRNA editing efficiency, with deep sequencing via Hi-TOM (http://www.hi-tom.net/hi-tom/index-CH.php).^24^ The results revealed no significant off-target effects (Figure 1C).

### Biochemical diagnosis of X-ALD

**Figure 2:**
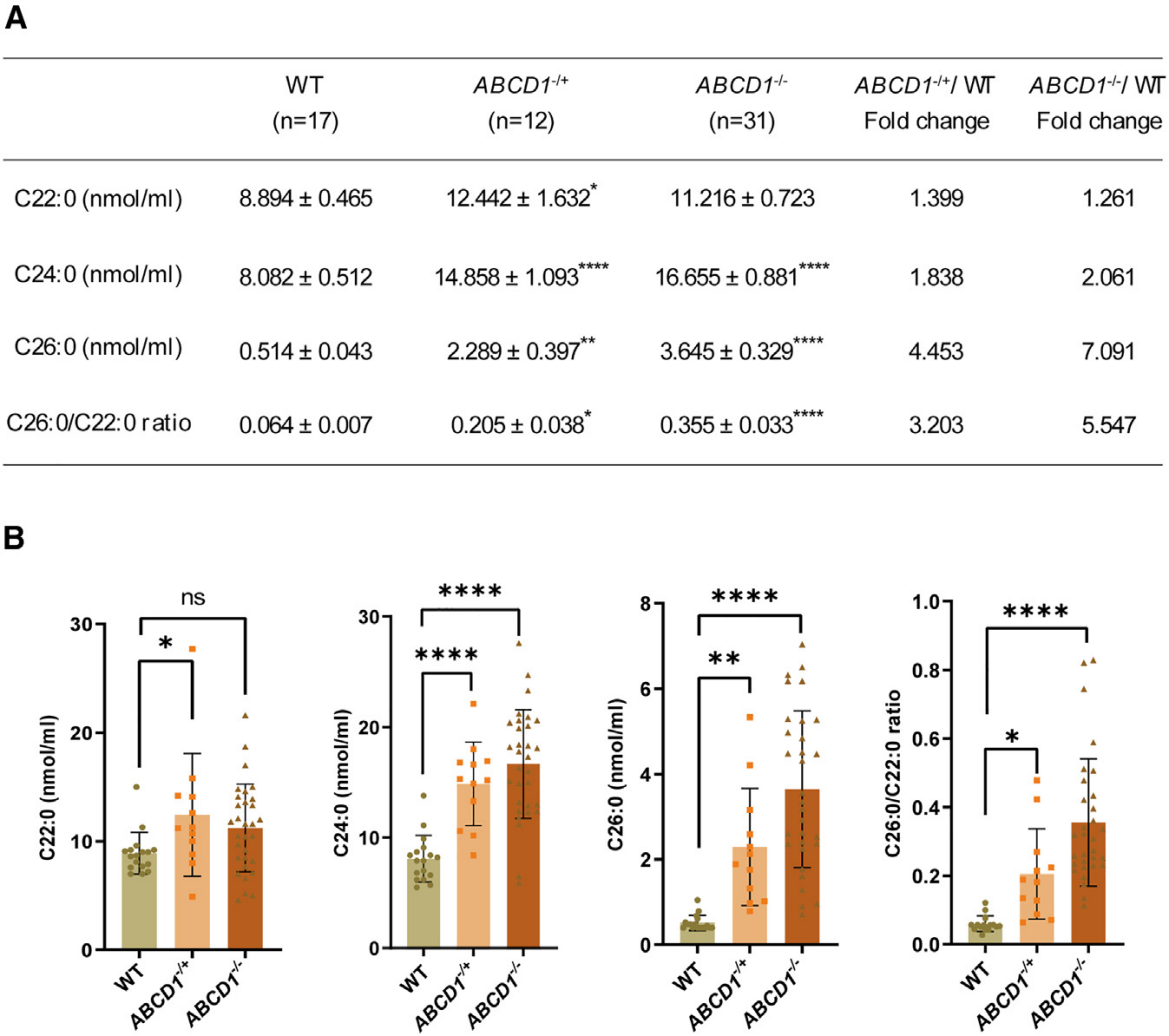
Serum VLCFA levels in WT and *ABCD1*-mutated rabbits (A) Table of the VLCFAs content in serum. Values are presented as mean (SD). (B) Bar graph illustration of the VLCFA levels in serum. ∗*p* < 0.05, ∗∗*p* < 0.01, ∗∗∗*p* < 0.001, ∗∗∗∗*p* < 0.0001, by one-way ANOVA, followed by Dunnett’s multiple comparisons test.

Serum samples were collected from WT (*n* = 17) and *ABCD1*-mutated rabbits (*ABCD1*^*−/+*^ *n* = 12; *ABCD1*^*−/−*^ *n* = 31) to measure VLCFA levels. Compared with WT, C24:0 and C26:0 levels were significantly elevated by 2.1-fold (16.655 ± 0.881 vs. 8.082 ± 0.512 nmol/mL) and 7.1-fold (3.645 ± 0.329 vs. 0.514 ± 0.043 nmol/mL) in *ABCD1*^*−/−*^ rabbits, respectively. *ABCD1*^*−/+*^ rabbits showed increases of 1.8-fold (14.858 ± 1.093 vs. 8.082 ± 0.512 nmol/mL) for C24:0 and 4.5-fold (2.289 ± 0.397 vs. 0.514 ± 0.043 nmol/mL) for C26:0. A significant rise in serum C22:0 was noted only in *ABCD1*^*−/+*^ rabbits (12.442 ± 1.632 nmol/mL) compared with WT (8.894 ± 0.465 nmol/mL), while *ABCD1*^*−/−*^ levels were 11.216 ± 0.723 nmol/mL. The average C26:0/C22:0 ratio showed significant increases of 5.5-fold (0.355 ± 0.033 vs. 0.064 ± 0.007 nmol/mL) in *ABCD1*^*−/−*^ and 3.2-fold (0.205 ± 0.038 vs. 0.064 ± 0.007 nmol/mL) in *ABCD1*^*−/+*^compared with WT (Figure 2). Biochemical evidence indicates successful disruption of the *ABCD1* gene in the mutant rabbits, leading to excessive accumulation of C24:0 and C26:0 in their blood starting at 3 months of age.

### Brain and spinal cord MRI and ultrastructural observation

**Figure 3:**
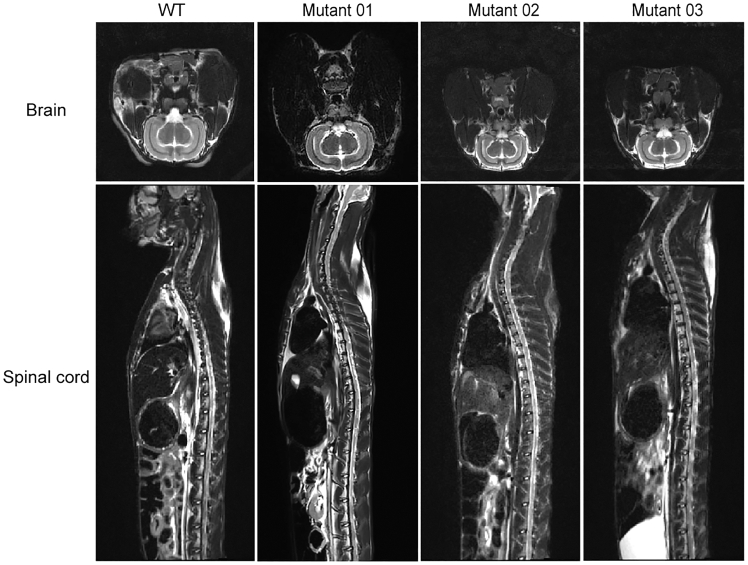
T2-weighted MRI of WT and *ABCD1*^*−/−*^ rabbit brains and spinal cords

**Figure 4:**
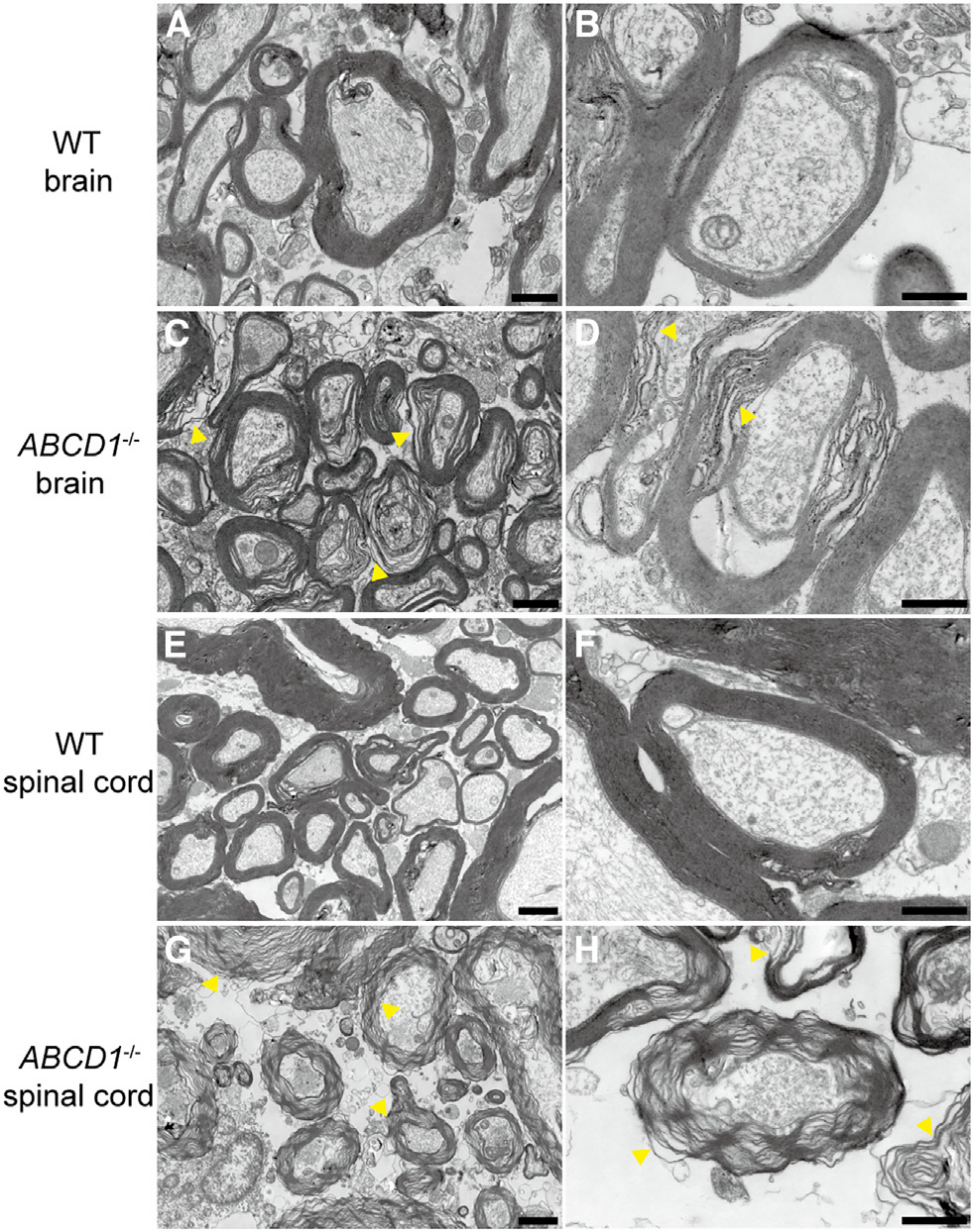
Ultrastructure morphology of WT and *ABCD1*^*−/−*^ rabbit brains and spinal cords Normal myelin structure is seen in the white matter of the brain (A and B) and spinal cord (E and F) of WT rabbits, while obvious demyelination is observed in the white matter of the brain (C and D) and spinal cord (G and H) of *ABCD1*^*−/−*^ rabbits. Yellow arrowheads indicate demyelination. Scale bar, 1 μm for (A) and (C); 2 μm for (E) and (G); 0.5 μm for (B), (D), (F), and (H).

T2-weighted MRI was conducted to evaluate neurological changes in the CNS of *ABCD1*^*−/−*^ rabbits. Contrary to expectations of demyelination, no symmetric hyperintensities were observed in the corpus callosum or parieto-occipital areas up to 21 months. MRI scans revealed normal neuronal patterns in both the brain and spinal cord (Figure 3). To further confirm whether there was neuropathy in the brain and spinal cord, we performed ultrastructural observation of the tissues through transmission electron microscopy (TEM). Abundant myelinated nerves with no obvious atrophy were observed, and the mitochondria were normal in the white matter of both WT and *ABCD1*^*−/−*^ rabbits at 24 months old. Notably, distinct demyelination was observed in the brain and spinal cord white matter of the *ABCD1*^*−/−*^ rabbits. The lamellar structure was disordered in the spinal cord white matter of *ABCD1*^*−/−*^ rabbits, contrasting with the normal myelin structure seen in WT rabbits (Figure 4). These findings suggest that 24-month-old *ABCD1*^*−/−*^ rabbits exhibit demyelination or spinal cord neurological lesions.

Observationally, *ABCD1*^*−/−*^ rabbits displayed several behavioral changes or symptoms typical of X-ALD, such as muscle weakness, inactivity, and dullness compared with the WT group (Video S1). Based on these results and observations, we conclude that our *ABCD1*^*−/−*^ rabbits manifested biochemical defects and neurological deterioration resembling CALD, the severe type of human X-ALD.

Video S1. Behavioral Characteristics of *ABCD1*^−/−^ rabbit *ABCD1*^−/−^ rabbits displayed several behavioral changes or symptoms typical of X-ALD, such as muscle weakness, inactivity, and dullness.

### Construction of rAAV9-h*ABCD1* vector

The rAAV9-h*ABCD1* vector was packaged using three plasmids: rAAV9-h*ABCD1*/rAAV9-*eGFP*, adenovirus helper, and rep2/cap9. Transfection efficiency exceeded 90% in 293FT cells expressing GFP (Figures S1A and S1B). After 72 h, rAAV9 was collected, confirming high efficiency. For large-scale production, rAAV9-h*ABCD1* was concentrated using POROS CaptureSelect™ AAV Resins following purification. The virus titer, measured by qPCR, excluded empty capsids, employing h*ABCD1* plasmid serial dilutions for absolute quantification (Figure S1C). SDS-PAGE confirmed the presence of capsid proteins VP1, VP2, and VP3 at 87 kDa, 72 kDa, and 62 kDa, respectively (Figure S1D).

### rAAV9-h*ABCD1* therapy

**Figure 5:**
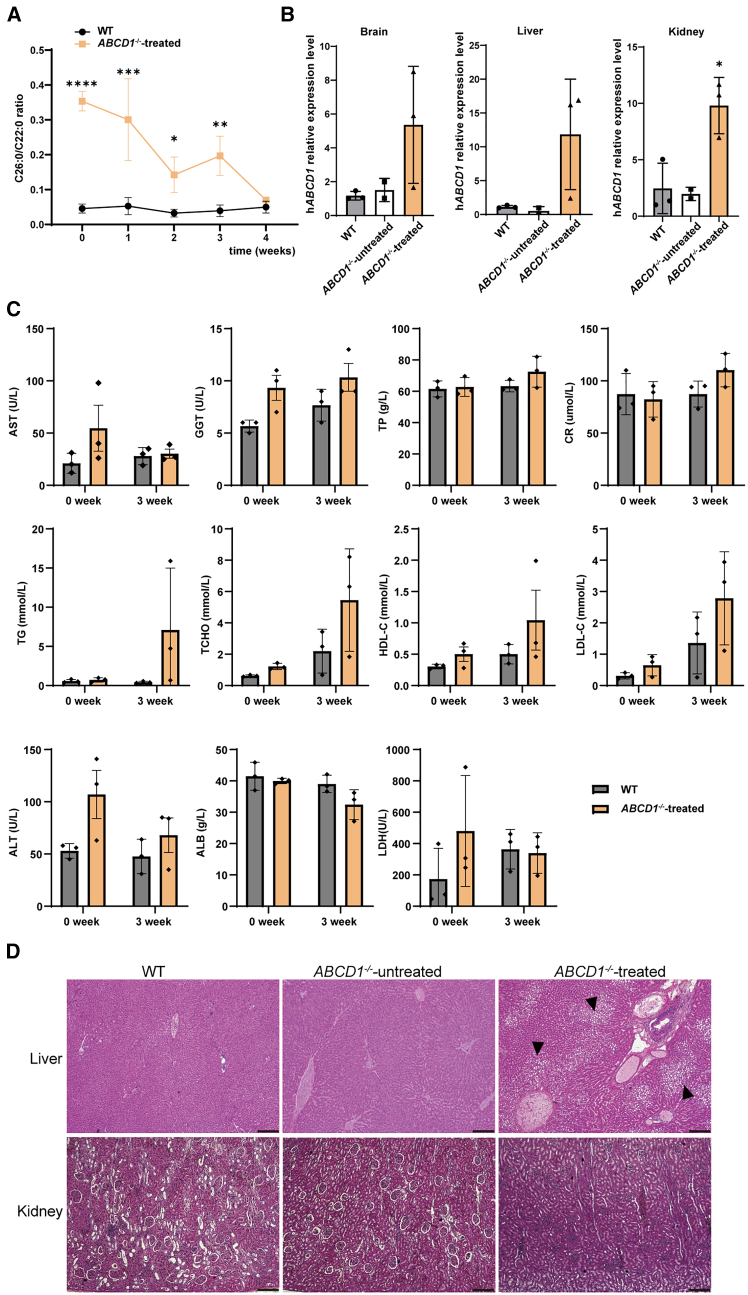
Effect of rAAV9-h*ABCD1* treatment on mutant rabbits (A) Monitoring VLCFA (C26:0/C22:0 ratio) levels post rAAV9-*hABCD*1 gene therapy. Two-way ANOVA followed by Bonferroni’s multiple comparisons test. Values presented as means (SD), ∗*p* < 0.05, ∗∗*p* < 0.01, ∗∗∗*p* < 0.001, ∗∗∗∗*p* < 0.0001. (B) Expression of *hABCD1* in different tissues among different groups determined by qPCR. ∗*p* < 0.05, by one-way ANOVA followed by Dunnett’s multiple comparisons test. (C) Blood biochemical levels before and after treatment in the rAAV9-h*ABCD1* group compared with the WT group. AST, aspartate aminotransferase; GGT, gamma-glutamyl transferase; TP, total protein; CR, creatinine; TG, triacylglycerol; TCHO, total cholesterol; HDL-C, high-density lipoprotein cholesterol; LDL-C, low-density lipoprotein cholesterol; ALT, alanine transaminase; ALB, albumin; LDH, lactate dehydrogenase. (D) Liver and kidney pathologies in rAAV9-h*ABCD1*-treated *ABCD1*^*−/−*^ rabbits, untreated *ABCD1*^*−/−*^ mutants, and the WT group. The black arrow indicates the lipid droplet vacuole. Scale bar, 250 μm.

In the rAAV9-h*ABCD1* therapy study, three *ABCD1*^*−/−*^ rabbits were administered 1 × 10^14^ V.G./kg of rAAV9-h*ABCD1*, while WT rabbits received 1 × 10^14^ V.G./kg of rAAV9-*eGFP* as a control. To assess the functionality of intravenously delivered rAAV9-h*ABCD1*, serum samples were collected weekly for a month. Prior to treatment, the mean C26:0/C22:0 ratio in the mutant group was significantly higher than that in the WT group. Two weeks post-injection, the ratio decreased by 59.7% in the mutant group. Excitingly, after 3 weeks of rAAV9 injection, the C26:0/C22:0 level declined to a comparable level, showing a notable difference (Figure 5A). Unfortunately, mutant rabbits developed foot inflammation and infection due to their environment, limiting monitoring to 4 weeks only.

Tissues from the brain, kidney, and liver were harvested to assess *hABCD1* expression via qPCR, revealing higher *ABCD1* levels in different tissues of the treated *ABCD1*^*−/−*^ rabbits compared with the untreated *ABCD1*^*−/−*^ group and WT group (Figure 5B). The expression level of hABCD1 protein in the brain of treated ABCD1^−/−^ rabbits was higher than that in other groups, despite hABCD1 signals being detected in the untreated *ABCD1*^*−/−*^ group and WT group, as rabbit ABCD1 is 94% homologous to human ABCD1 (Figure S1E). This indicates the successful passage of rAAV9-h*ABCD1* across the BBB into brain cells.

To assess potential side effects of rAAV9-h*ABCD1*-based gene therapy, blood biochemical tests and hematoxylin and eosin (H&E) staining were performed and compared between treated *ABCD1*^*−/−*^ rabbits and the WT group. Prior to treatment, serum levels of aspartate aminotransferase (AST), alanine aminotransferase (ALT), gamma-glutamyl transferase (GGT), total cholesterol (TCHO), high-density lipoprotein cholesterol (HDL-C), low-density lipoprotein cholesterol (LDL-C), and lactate dehydrogenase (LDH) were slightly elevated in the treatment group compared with the WT group. Three weeks after rAAV9 injection, GGT, TCHO, HDL-C, and LDL-C increased in both groups, while total protein (TP), creatinine (CR), and triacylglycerol (TG) increased only in the treatment group, with albumin (ALB) and ALT decreasing. Nonetheless, these changes were not statistically significant (Figure 5C). Histopathological examination revealed no pathological differences in kidney tissue among treated *ABCD1*^*−/−*^ rabbits, untreated *ABCD1*^*−/−*^ rabbits, and the WT group. However, hepatic steatosis was observed in the livers of treated *ABCD1*^*−/−*^ rabbits, while no lipidosis was present in untreated *ABCD1*^*−/−*^ rabbits or the WT group (Figure 5D).

Overall, these results indicate that rAAV9-h*ABCD1* gene therapy can effectively lower VLCFA levels and transduce the h*ABCD1* gene into brain cells without significant side effects. Nonetheless, monitoring was prematurely curtailed due to an unexpected foot infection related to the environment. Further investigation into the long-term effects of rAAV9-h*ABCD1* treatment is needed to fully evaluate its effectiveness and safety over time.

## Discussion

X-ALD has a devastating impact, leading to fatalities and imposing a significant burden on patients' families and society.^2^^,^^25^ The X-ALD database (https://adrenoleukodystrophy.info/) catalogs over 800 non-recurrent *ABCD1* mutations, with 49% being missense mutations.^23^ While phenotypic variability in X-ALD is influenced by multiple factors, the *ABCD1* gene is the most extensively studied in terms of its pathophysiology. Approximately 1 in 14,700 live births manifest X-ALD, with 95% of affected individuals being male, and most females being heterozygotes. Although newborn screening for X-ALD is gradually being adopted in some countries, many patients remain untreated.^26^^,^^27^

The pathogenesis of X-ALD encephalopathy remains incompletely understood. Although the mutant *ABCD1* gene itself is not neurotoxic, the accumulation of VLCFA in various tissues such as the brain, spinal cord, peripheral nerves, adrenal glands, and testicles is reported to induce CNS toxicity, oxidative stress, and apoptosis, forming the underlying pathological process.^28^ Both endogenous and exogenous VLCFAs can cause tissue damage. Accumulated VLCFAs disrupt the integrity of the plasma membrane, resulting in cell toxicity and death, specifically in oligodendrocytes in the neural system, which is the sole cause of X-ALD in patients. The accumulation of VLCFAs provokes damage to oligodendrocytes, thereby developing demyelination in the white matter of the brain and spinal cord. Additionally, cholesterol esterified with VLCFAs contributes to adrenocortical insufficiency in X-ALD.^29^ The complex genotype-phenotype relationship complicates disease prediction and monitoring, necessitating regular VLCFA detection and MRI assessments.^30^^,^^31^^,^^32^

The most severe form of X-ALD, CALD, currently has no cure other than early-stage bone marrow transplantation, highlighting the need for innovative therapies. HSCT can alleviate symptoms if performed early; however, its effectiveness diminishes in later stages, increasing the risk of serious fatal complications. Some childhood HSCT patients developed AMN in early adulthood,^33^ suggesting that clearing circulating VLCFAs may not fully resolve the disease. Herein, we believe that targeting the brain, the most affected area, might be a more effective strategy for many CALD patients. A recent Phase I clinical trial has reported favorable results from intracerebral lentiviral-*ABCD1* injections.^34^ While efficacy in this trial was limited, likely due to the advanced disease stages of participants, intracerebral gene correction remains a promising approach despite its invasiveness.

The rAAV vector, delivered via lumbar cerebrospinal fluid in *Abcd1*^*−/−*^ mice, has shown potential in reducing VLCFA levels, indicating a path for AAV-mediated gene therapy.^35^ Given the systemic effects of X-ALD on various tissues, intravenous injection of rAAV, known for its broad tissue tropism and ability to cross the BBB, emerges as a promising treatment strategy.^36^^,^^37^^,^^38^ AAV9 has long been reported to have a promising ability to cross the BBB, and its variant AAV-PHP.B was reported to have 40-fold higher efficiency than AAV9 in C57BL/6J mice.^39^ However, the BBB permeability across different genetic backgrounds and species remains unclear. Some studies showed that AAV-PHP.B exhibits high CNS transduction in some mouse strains such as C57BL/6J, SJL/J, FVB/N, and DBA/2, but it cannot transduce in the brain in BALB/c.^40^ Moreover, the BBB-crossing efficiency was found to be low in marmosets and rhesus macaques.^41^ Considering the uncertain BBB permeability of AAV-PHP.B in rabbits, we opted to use AAV9 for our studies.

Although multiple *Abcd1*-deficient mouse models have been reported to recapitulate X-ALD, they do not spontaneously present cerebral pathology like humans with CALD.^21^ Besides, due to the differences in physiological features and gene expression between mice and humans, the short lifespan of mice is unsuitable for longitudinal studies on the safety and efficacy of therapeutic strategies. Currently, the lack of a reliable X-ALD animal model has hindered thorough investigations into the disease’s pathophysiology. Compared with mice or rats, rabbits are closer to humans in terms of physiology, anatomy, and genetics. They are also cheaper to raise and have a shorter gestation period than pigs or monkeys. Rabbits are widely used as models in research on human cardiovascular and metabolic diseases.^42^ Our study addresses this by introducing a novel *ABCD1*^−/−^ rabbit model via CRISPR-Cas9 technology. The phenotypic characteristic of elevated VLCFA levels in blood and demyelination in the brain and spinal cord demonstrates the promising potential of this model for studying X-ALD pathophysiology and molecular mechanisms.

Prior research on direct intracerebral lentiviral *ABCD1* injections in ALD knockout mice suggests potential dosage reduction strategies.^4^ Optimizing rAAV9-h*ABCD1* dosage and infusion methods is crucial for sustained therapeutic effects while considering clinical feasibility. Exploration of rAAV9-based gene therapy in this rabbit model involved intravenous injection of rAAV9-h*ABCD1*, which successfully penetrated the BBB, resulting in a short-term reduction of VLCFA levels. The transcriptional and protein expression levels of gene h*ABCD1* in the *ABCD1*^−/−^-treated group also proved the successful delivery of rAAV9-h*ABCD1* to brain cells (Figures 5B and S1E). However, the antibodies used are not specific enough to recognize the target protein and may result in the appearance of non-specific bands (Figure S1E). We assume that the signal in the *ABCD1*^−/−^-untreated sample is due to non-specific binding. Unfortunately, unexpected deaths have limited promising outcomes, necessitating further investigation into long-term effects.

Notably, while blood biochemical parameters remained stable post-treatment with rAAV9-h*ABCD1* (Figure 5C), identified lipid droplet vacuoles in the liver raise concerns about hepatic metabolic disorders (Figure 5D). The liver is the primary site for lipid metabolism, where neutral lipid accumulation can lead to lipid droplet formation in hepatocytes. It has been shown that fatty acids with carbohydrate chains (C12–C18) induce lipid droplet formation in a hepatocyte cell line. Long-chain fatty acids, once esterified into neutral lipid molecules like triacylglycerol (TG), are stored in liver cells and can lead to droplet formation.^43^ Our findings indicate hepatic steatosis in the *ABCD1*^−/−^-treated group, contrasting with the WT and untreated *ABCD1*^−/−^ group. Furthermore, 3 weeks post-treatment, TG and TCHO levels increased in the blood of the treated *ABCD1*^−/−^ group. We hypothesize that the synthesis of normal ABCD1 protein promotes the clearance of accumulated VLCFAs from the blood, leading to a significant short-term spike in free long-chain fatty acids, which are subsequently transported to the liver, causing lipid droplet formation.

In summary, despite existing challenges, rAAV-based gene therapy shows significant promise for treating X-ALD. Ongoing technological advancements and in-depth research are anticipated to establish rAAV-based gene therapy as a critical and effective treatment for X-ALD. Our novel rabbit model significantly enhances the array of available research animal models, contributing valuable insights into X-ALD pathophysiology, therapeutic method development, and gene therapy applications.

## Materials and methods

### Generation of *ABCD1*-mutated rabbit model

The *ABCD1*-mutated rabbits were generated in Guangzhou by Dr. Liangxue Lai’s research team at the Guangzhou Institutes of Biomedicine and Health, Chinese Academy of Sciences. Female New Zealand White rabbits aged 6–8 months were administered 50 IU follicle-stimulating hormone six times every 12 h to induce superovulation. Following mating, the rabbits received an injection of 100 IU human chorionic gonadotropin and were euthanized 18 h post-injection. Rabbit embryos at the pronuclear stage were collected by flushing the oviducts with 5 mL DPBS-BSA. Subsequently, 200 ng/μL Cas9 mRNAs and 50 ng/μL sgRNAs were mixed and microinjected into the rabbit zygotes. The embryos were cultured in Earle’s Balanced Salt Solution at 38.5°C in a 5% CO_2_ incubator and then transferred into the oviduct of a female rabbit to generate the *ABCD1*-mutated offspring. Two sgRNAs were designed to target the first exon of *ABCD1*. The sgRNA sequences were: 5′- GGCGGGCCACATACACCGAC -3′, and 5′- CTGCCAGCTGAACGCCCGTG-3’.

### Genotyping of rabbits

Genomic DNA was isolated from rabbit ear tissue using a DNA extraction kit (Qiagen, #51304). The DNA concentration was measured using a Nanodrop Spectrophotometer (Nanodrop Technologies, #ND-1000). Extracted DNA was subjected to polymerase chain reaction (PCR) using h*ABCD1* primers (Table S1). PCR products were purified by PCR Purification Kit (Qiagen, #28106) and sent for Sanger sequencing.

### Magnetic resonance imaging

To facilitate anesthesia, a mixture of 50 mg ketamine and 10 mg xylazine per kg body weight was intramuscularly injected into the rabbit thigh. Continuous monitoring of heart rate and breathing rate occurred until the rabbit regained consciousness. T2-weighted imaging was performed to analyze the neurological changes.

### Transmission electron microscopy

The brain and spinal cord white matter were cut into 1- to 3-mm^3^ sections immediately within 1–3 min and fixed in the TEM fixative at 4°C overnight, while keeping vacuum extraction until the samples sank to the bottom. The sectioned tissues were then osmicated with 1% osmic acid (O_S_O_4_) in 0.1M phosphate buffer (PB, pH 7.4) for 2 h at room temperature, avoiding light. O_S_O_4_ was removed and the tissues were washed with 0.1M PB for 15 min, three times. After stepwise dehydration, the tissue sections were embedded in a resin mix and polymerized at 60°C for 48 h. The resin blocks were cut into 70-nm thin sections on the ultra-microtome and the tissues were fished out onto 150-mesh copper grids with formvar film. The tissues were stained with 2% uranium acetate saturated alcohol solution, avoiding light staining for 8 min, rinsed in 70% ethanol three times, and then rinsed in ultra-pure water three times. They were then stained with 2.6% lead citrate, avoiding CO_2_ staining for 8 min, and rinsed with ultra-pure water three times. The copper grids were dried overnight at room temperature. Observation was conducted under a transmission electron microscope (HT7800 RuliTEM, Hitachi, Japan) and images were captured.

### Construction of rAAV-h*ABCD1* plasmid

The recombinant adeno-associated virus serotype 9 (rAAV9) plasmid, graciously provided by Prof. Patrick Aubourg of INSERM, France, served as the foundational element. The human *ABCD1* (h*ABCD1*) gene was meticulously amplified from plasmid pRRL-MND-h*ABCD1* via PCR. The resulting 2-kb h*ABCD1* fragment, flanked by MluI-HF and XhoI restriction sites, was obtained. The rAAV9 vectors and h*ABCD1* fragments were subjected to digestion by MluI-HF and XhoI enzymes in CutSmart® Buffer (New England Biolabs, #B7204S) under standard conditions (37°C overnight). The separated fragments underwent gel electrophoresis, with the smaller fragments being isolated and purified via a Qiagen gel extraction kit (#28704). Ligation of the purified products was performed with T4 DNA Ligase (New England Biolabs, #M0202S) under specified conditions (16°C for approximately 12 h). The resultant ligation product was transformed into competent *E. coli* cells via standard heat-shock transformation procedures. Successful colonies were screened to extract the rAAV9-h*ABCD1* plasmids, which underwent sequencing to confirm the absence of mutations. Additionally, rAAV9-*eGFP* plasmids were constructed using identical methodologies to gauge transduction efficiency.

### Packaging of rAAV9 vectors

293FT cells were seeded into several 15-cm^2^ culture dishes in Dulbecco’s Modified Eagle’s Medium DMEM-High Glucose (DMEM-HG) (Hyclone, #SH30022.02) supplemented with 10% fetal bovine serum (FBS) (Hyclone, #SV30160.03). At around 80% confluency, the medium was changed to DMEM-HG supplemented with 5% FBS at least 2 h before transfection. To prepare the transfection mixture, packaging plasmids were added to polyethyleneimine (PEI, Polysciences, Inc., #23966) in a 1:3 ratio, total plasmid mass (mg) to PEI volume (μL), in a total of 2 mL Opti-MEM™ Reduced Serum Medium (Gibco™, #31985070). For example, 1 mL of medium containing Adenohelper plasmid (15 mg), rep2/cap9 plasmid (15 mg), and rAAV9-h*ABCD1* plasmid/rAAV9-*eGFP* plasmids (30 mg) were added to 1 mL of medium containing 180 μL of PEI. After incubation for 20 min at room temperature, the mixture was added to the 15-cm^2^ plate of cells and mixed gently. Cells were incubated in a 5% CO_2_ incubator at 37°C for 16 h. After 16 h post-transfection, the medium was changed to fresh DMEM-HG supplemented with 5% FBS and again incubated in a 5% CO_2_ incubator at 37°C for 56 h. At 72 h post-transfection, medium and cell pellets were collected as they contained AAV particles. The bottles were centrifuged at 4000 rpm for 15 min to pellet the cells. The supernatant was filtered through 0.22-μm syringe filters and prepared for affinity purification. The cell pellet was resuspended in 20–30 mL of AAV Lysis Buffer and transferred to 50-mL Falcon tubes for cell lysis. The lysate was frozen and thawed in liquid nitrogen four to six times to reduce the viscosity from genomic DNA. The cold lysate was sonicated until the DNA was fragmented. The lysate was centrifuged at 25,000 × *g* for 30 min, repeating this process two to three times until the supernatant was clear. The lysate supernatant was filtered through 0.22-μm syringe filters, and the rAAV9 viruses can be high-yield through a POROS CaptureSelect™ AAV Resins. A chromatography column was filled with 1 mL of resins, then rinsed with >10 column volumes (CVs) of wash buffer. The resin was rinsed again with >10 CVs of PBS, and the clarified media supernatant was poured into the column. AAV was eluted by adding glycine elution buffer (1 mL at a time) to the resin and allowed to drip into tubes containing 1 M Tris (PH 8). The A260/280 was checked, and peak fractions were collected. The eluted medium was transferred to a Centricon column with a 100-kDa cutoff. Centrifugation and buffer exchange were performed by resuspending the concentrated AAV in PBS +0.01% Pluronic F-68. Centrifugation was repeated to concentrate the AAV at least two times to obtain highly purified rAAV9.^44^ The rAAV9 titers were determined by quantitative PCR (qPCR) using SYBR Green Premix Taq (TakaRa, #RR820) according to the manufacturer’s instructions and expressed as vector genomes per milliliter (V.G./mL) as described previously.^45^

### Intravenous injection of rAAV9 virus

The rAAV9-h*ABCD1* vectors were diluted in normal saline to a volume of 500 μL, carrying a dose of 1 × 10^14^ V.G./kg, before being injected into *ABCD1*^*−/−*^ rabbits intravenously through the ear vein. As a comparison, the same dose of rAAV9-*eGFP* was injected into age-matched WT rabbits intravenously through the ear vein using the same method. The sex of the rabbits was randomly selected, with two female and one male rabbit in each group (see Table S2 for details). All rabbits were 9 months old at the time of treatment.

### Ethics approval

All experimental procedures involving animals were conducted under the guidance of the Guide for Animal Welfare and Care in Guangdong Province and were approved by the Laboratory Animal Ethics Committee of Guangzhou Huateng Biomedical Technology Co., Ltd. (Ethics approval number: HTSW210513).

### Serum and tissue preparation

Blood was collected weekly from the rabbits’ ear veins, centrifuged at 3000 rpm for 20 min, and the serum was stored at −80°C. VLCFA levels in serum were measured using gas chromatography-mass spectrometry (GC-MS). After rabbits sacrifice, various tissues were snap-frozen and stored at −80°C. Some tissues were fixed in 4% paraformaldehyde and processed into paraffin blocks. The paraffin sections, 5 μm thick, were stored at room temperature and stained with H&E according to standard protocols.

### Statistics

An unpaired Student’s t test was used to compare two experimental conditions with normal distribution. One-way analysis of variance (ANOVA) followed by Dunnett’s multiple comparisons test was applied for comparisons of more than two groups. Two-way ANOVA followed by Bonferroni’s multiple comparisons test was used for groups with different genotypes and treatments as factors. All analyses were performed with GraphPad Prism software version 9.3.1. All data are presented as mean ± standard deviation (SD). A *p* value < 0.05 was considered statistically significant (∗*p* < 0.05, ∗∗*p* < 0.01, ∗∗∗*p* < 0.001, ∗∗∗∗*p* < 0.0001).

## Data and code availability

All data are available in the article. Requests for materials should be addressed to the corresponding author. Reprints and permissions information is available.

## Acknowledgments

This work was supported in part by the 10.13039/501100012166National Key Research and Development Program of China (2023YFA0914904 and 2023YFA0914900); 10.13039/501100017610Shenzhen Science and Technology Innovation Program (JCYJ20210324114606019); and the Start-up Grant for Stem Cell Regenerative Medicine (10.13039/100017696Guangzhou Women and Children's Medical Center, 10.13039/100009659Guangzhou Medical University, 5001-4001010). We deeply appreciate and memorialize Mr. Wanchou Lian (passed away in March 2022) for his dedication to take care of *ABCD1* knockout rabbits.

## Author contributions

Conceptualization, Supervision, Writing – review & editing: Q.L.; Writing – original draft: X.Z. (Xiaoya Zhou); Writing – review & editing: X.Z. (Xiaoya Zhou), C.M.; Investigation: X.Z. (Xiaoxian Zhang), X.X., Z.L. (Ziyue Li), J.L., J.W., M.K., S.C.; Methodology: F.D., L.C., L.G., H.C.; Resources: H.L., L.Z., Z.L. (Zhejin Li), T.S., Z.L. (Zhanjun Li), L.L., P.Y., Z.L. (Zhijian Lin). All authors read and approved the final manuscript.

## Declaration of interests

The authors declare no competing interests.

## Supplemental information

Document S1. Figure S1, Tables S1, and S2

Document S2. Article plus supplemental information
