# Supplementary material for: Development of a rabbit model for adrenoleukodystrophy: A pilot study on gene therapy using rAAV9
Source: Mol Ther Nucleic Acids. 2025 Feb 3;36(1):102469. doi: 10.1016/j.omtn.2025.102469 (PMC11872110; doi:10.1016/j.omtn.2025.102469)
Supplement: Document S1. Figure S1, Tables S1, and S2 [file mmc1.pdf]

OMTN, Volume *36*

Supplemental information

Development of a rabbit model for adrenoleukodystrophy: A pilot study on gene therapy using rAAV9

Xiaoya Zhou, Chui-Yan Ma, Xiaoxian Zhang, Xianchuan Xu, Fuyu Duan, Meng Kou, Hongsheng Liu, Liang Zeng, Liyan Guo, Shaoxiang Chen, Li Chen, Ziyue Li, Jie Luo, Jieying Wu, Zhejin Li, Zhanjun Li, Tingting Sui, Ping Yuan, Zhijian Lin, Hao Chen, Liangxue Lai, and Qizhou Lian

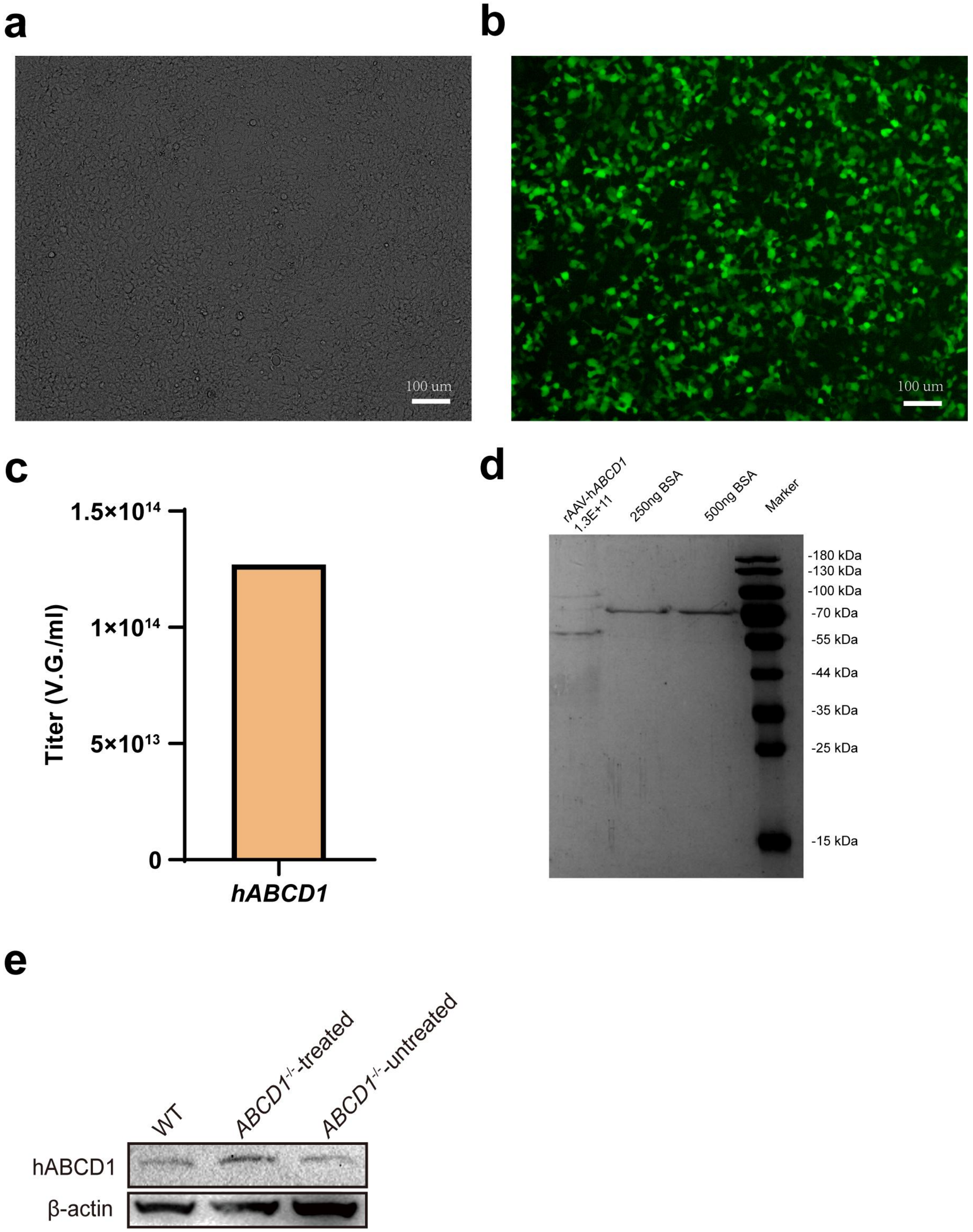

Figure S1. The transfection efficiency, virus titer, and purification of rAAV9-*hABCD1* and the expression evidence of hABCD1 in the brain tissue. a,b. Brightfield and GFP channel of 293FT cells expressing eGFP in 72 hours post-transfection in rAAV-*eGFP* controls. Over 90% of cells were successfully transfected with *eGFP* plasmid. The presence of green fluorescent cells showed successful production of functional rAAV-*eGFP*. c. The virus titer of

rAAV9-h*ABCD1*. d. SDS-PAGE separated protein extracts of purified rAAV9-h*ABCD1. e.* Expression of hABCD1 in brain tissues among different groups determined by Western blot.

Table S1. Details of primers.

**Primers**	
**For rabbit genotyping**	
ABCD1 Forward	CTGGCATGAACCGAGTCTTC
ABCD1 Reverse	GCTTGGTCAGGTTGGAGTAG
**For h*ABCD1* gene amplification**	
h*ABCD1* Forward	CGCAGGCGTATGCCGGTGCTCTAAAGGC
h*ABCD1* Reverse	GGTCTCGAGTCAGGTGGAGGCACCCTGG
**For virus tittering qPCR**	
rAAV9-h*ABCD1* Forward	TCGTCAACAGTGCCATCCGT
rAAV9-h*ABCD1* Reverse	CGAAGCAGGGTGTAGGAAATCA
**For relative quantification of gene expression**	
h*ABCD1*-specific Forward	CCCTCCTCGTCAGTCTCTCA
h*ABCD1*-specific Reverse	TGGGATCTTTGGGGCTCTTG
GAPDH Forward	ATGTTCGTCATGGGTGTGAA
GAPDH Reverse	GGTGCTAAGCAGTTGGTGGT
**For knockdown**	
sgRNA1	GGCGGGCCACATACACCGAC
sgRNA2	CTGCCAGCTGAACGCCCGTG
**For off-target detection**	
sgR1-OT1J Forward	GGAGTGAGTACGGTGTGCGGGCAAGGAGGACACTAATC
sgR1-OT1J Reverse	GAGTTGGATGCTGGATGGACGTGACTGATCTTGCACTTG
sgR1-OT2J Forward	GGAGTGAGTACGGTGTGCGATTATACCCATAGGTCCCACAG

sgR1-OT2J Reverse	GAGTTGGATGCTGGATGGTCAGAGATAGTTTGCAGAAGAGAA
sgR1-OT3J Forward	GGAGTGAGTACGGTGTGCCACGTGGTAGTTCCGTTCAT
sgR1-OT3J Reverse	GAGTTGGATGCTGGATGGGTAGCTCTGGTGGTCCCT
sgR1-OT4J Forward	GGAGTGAGTACGGTGTGCGAAGGCTGGTGTTGGTGATA
sgR1-OT4J Reverse	GAGTTGGATGCTGGATGGAGCTAGTGGGAGGGTGATTA
sgR1-OT5J Forward	GGAGTGAGTACGGTGTGCGCTCTGACCATCCAGCA
sgR1-OT5J Reverse	GAGTTGGATGCTGGATGGATCTGGAACAAGGCACCA
sgR1-OT6J Forward	GGAGTGAGTACGGTGTGCCCACATGGTGTGTGGTGAG
sgR1-OT6J Reverse	GAGTTGGATGCTGGATGGCAGGCAAACTAAGGCCAGAA
sgR2-OT1J Forward	GGAGTGAGTACGGTGTGCAAGTAGTATCTGGTCACCACTTTG
sgR2-OT1J Reverse	GAGTTGGATGCTGGATGGAAAGCAAGTCCCATGAGTCC
sgR2-OT2J Forward	GGAGTGAGTACGGTGTGCCCACAGGGAACCTAAGAGATG
sgR2-OT2J Reverse	GAGTTGGATGCTGGATGGAGTTGGGCAAAGTTACCAATTT
sgR2-OT3J Forward	GGAGTGAGTACGGTGTGCAGAAGAATCCTGCTCGTTTCG
sgR2-OT3J Reverse	GAGTTGGATGCTGGATGGGCTCAGCAGCCAATCACA
sgR2-OT4J Forward	GGAGTGAGTACGGTGTGCCTGAACTTGGAGCATGGGAAA
sgR2-OT4J Reverse	GAGTTGGATGCTGGATGGAGGAGAGGTCTCAGTGTTCTG
sgR2-OT5J Forward	GGAGTGAGTACGGTGTGCGTTGTCGCAATGCGTAAGTAAA
sgR2-OT5J Reverse	GAGTTGGATGCTGGATGGGTGGCTCTGTGGATGAGAAG
sgR2-OT6J Forward	GGAGTGAGTACGGTGTGCGCTTTGGTCTTTGCTTACCTTC
sgR2-OT6J Reverse	GAGTTGGATGCTGGATGGGCTTCTCATGCTTTCAGTTCAC

Table S2. Details of injection.

No.	Genotype	Gender	Age	Weight （kg）	Injection drugs	Total vector doses(V.G.)	Virus titer (V.G./ul)	Volume of vector (ul)	Volume of saline (ul)
1	Wide-type	Male	9 months	2.865	1×10^{14}vg/kg rAAV9_CAG_*eGFP*	2.87E+14	1.50E+12	191.0	309.0
2	Wide-type	Female	9 months	2.714	1×10^{14}vg/kg rAAV9_CAG_*eGFP*	2.71E+14	1.50E+12	180.9	319.1
3	Wide-type	Female	9 months	3.1	1×10^{14}vg/kg rAAV9_CAG_*eGFP*	3.10E+14	1.50E+12	206.7	293.3
4	*ABCD1*$^{-/-}$	Male	9 months	3.483	1×10^{14}vg/kg rAAV9_CAG_h*ABCD1*	3.48E+14	1.10E+12	316.6	183.4
5	*ABCD1*$^{-/-}$	Female	9 months	4.515	1×10^{14}vg/kg rAAV9_CAG_h*ABCD1*	4.52E+14	1.10E+12	410.5	89.5
6	*ABCD1*$^{-/-}$	Female	9 months	3.452	1×10^{14}vg/kg rAAV9_CAG_h*ABCD1*	3.45E+14	1.10E+12	313.8	186.2

Video S1. Behavioral Characteristics of *ABCD1*$^{-/-}$ rabbit. *ABCD1*$^{-/-}$ rabbits displayed several behavioral changes or symptoms typical of X-ALD, such as muscle weakness, inactivity, and dullness.
